# Global Health Partnerships During the COVID-19 Pandemic: Perspectives and Insights from International Partners

**DOI:** 10.4269/ajtmh.21-0156

**Published:** 2021-06-28

**Authors:** Megan S. McHenry, Reena P. Tam, Amira A. Nafiseh, Mary Ann Etling, Adelaide E. Barnes, Amy R. L. Rule, Heather L. Crouse, Heather Haq, Lee E. Morris, Brittany L. Murray, Lisa A. Umphrey, Elizabeth M. Keating

**Affiliations:** 1 Division of Pediatric Infectious Disease and Global Health and Director of Pediatric Global Health Education within the Department of Pediatrics, Indiana University School of Medicine, Indianapolis, Indiana;; 2 Division of Pediatric Hospital Medicine, Department of Pediatrics, University of Utah School of Medicine, Salt Lake City, Utah;; 3 Indiana University School of Medicine, Indianapolis, Indiana;; 4 Section of Hospital Medicine and Associate Program Director of the Pediatrics Residency Program at The Children’s Hospital of Philadelphia, Philadelphia, Pennsylvania;; 5 Divisions of Hospital Medicine and Perinatal Institute, Cincinnati Children’s Hospital Medical Center and University of Cincinnati College of Medicine, Cincinnati, Ohio;; 6 Department of Pediatrics at Baylor College of Medicine, Houston, Texas;; 7 Baylor College of Medicine International Pediatric AIDS Initiative (BIPAI) at Texas Children’s Hospital, Houston, Texas;; 8 Division of Pediatric Infectious Diseases and Immunology, Department of Pediatrics, Atrium Health Levine Children’s Hospital, Charlotte, North Carolina;; 9 Departments of Pediatrics and Emergency Medicine at Emory University School of Medicine, Atlanta, Georgia;; 10 Center for Global Health, Colorado School of Public Health, Denver, Colorado;; 11 Division of Pediatric Emergency Medicine, Department of Pediatrics, University of Utah, Salt Lake City, Utah

## Abstract

Global health partnerships (GHPs) have encountered many challenges during the coronavirus disease 2019 (COVID-19) pandemic. New perspectives and insights are needed to guide GHPs when navigating current and future collaborations. This study aimed to understand perspectives and insights of international partners regarding how the COVID-19 pandemic impacted their GHPs with institutions in the United States. We performed a cross-sectional qualitative study conducted through virtual semi-structured interviews performed between June 12, 2020 and July 22, 2020. We queried academic institutions based in the United States to refer individuals from their corresponding international GHP organizations. We invited these individuals to participate in virtual interviews that were audio-recorded and transcribed. We analyzed data qualitatively to identify themes. Eighty-four United States partners provided e-mail addresses for international partners. Ten individuals from these GHPs completed the interview. Participants reported overall positive experiences with their United States-based partners during the pandemic. The following themes emerged: imbalanced decision-making; worry about partnership continuity; opportunity to optimize communication within partnerships; interest in incorporating technology to facilitate engagement; and a desire for increased bilateral exchanges. Several challenges appeared to exist before COVID-19 and were highlighted by the pandemic. Most respondents were optimistic regarding the future of their GHPs. However, concerns were expressed regarding the implications of fewer in-person international experiences with United States trainees and the desire for stronger communication. Although our results do not represent the perspectives and insights of all GHPs, they provide considerations for the future. We urge institutions in the United States to re-examine and strive for equitable relationships with their international partners.

## INTRODUCTION

Global health partnerships (GHPs) between academic institutions based in the United States and international institutions have rapidly expanded in recent decades because of globalization, increased awareness of the economic connections to health, and the desire of medical professionals to be prepared to provide quality care for all.^1,2^ These academic GHPs aim to improve local healthcare capacity and increase knowledge among individuals within the partnership. Within these international collaborations, there is increasing movement toward ensuring equity and bi-directionality between institutions.^1–4^

The coronavirus disease 2019 (COVID-19) pandemic has disrupted GHPs. The need to prioritize local pandemic measures and travel restrictions have forced nearly all United States-based academic institutions to halt international efforts. Although a few institutions have used knowledge gained from their international partners to combat COVID-19 locally,^5^ many United States-based institutions missed opportunities to strengthen ties with their international partners during this time. Leaders in global health (GH) education based in the United States have discussed the considerations for returning to international travel,^6^ but little has been documented regarding international partner perspectives and their insights during the COVID-19 pandemic.

Before the pandemic, international partners provided valuable opinions about how to improve experiences for GH and local learners at their institutions.^7^ These perspectives and insights are critical for navigating this current challenging time. The objective of this study was to understand how the COVID-19 pandemic impacted GHPs between international and United States institutions. Through semi-structured interviews with international partners, we aimed to gain perspectives and insights regarding how GHPs can continue successful elements of their collaborations, how the pandemic highlighted any existing issues, and how to approach new challenges with respect and equity throughout the COVID-19 pandemic and beyond. By doing so, we believe that GHPs can create more meaningful collaborations for the future.

## MATERIALS AND METHODS

### Setting and participants.

We performed a cross-sectional qualitative study from June 2, 2020 to July 22, 2020. We queried GHPs from United States-based academic institutions via e-mail to refer individuals from their corresponding international partners. This e-mail described the study objectives and was sent through various GH listservs, including the American Academy of Pediatrics Section on Global Health, the Consortium of Universities for Global Health, and contact lists from the Academic Pediatric Program Directors Global Health Learning Community. These methods have been described elsewhere.^8^ E-mail addresses for international partners were requested. Prospective participants were contacted via e-mail or telephone to determine whether they fulfilled the following inclusion criteria: ability to speak English and represented an international institution partnered with a United States-based institution for GH training and international rotations. We excluded participants who were United States citizens representing international institutions because we wanted to prioritize perspectives directly from international partners. Individuals who fulfilled the criteria for the study were enrolled and a virtual interview was scheduled.

### Data collection.

Two authors (A. N., M. A. E.) trained in qualitative interviewing (by M. S. M.) performed the virtual interviews, which consisted of a study overview, verbal demographic survey, and semi-structured interview using an interview guide. The interviewers had no connection to the United States-based institutions or international partners recruited for the study to minimize desirability bias in responses. The author team comprising United States-based pediatric GH educators and members of the Academic Pediatric Program Directors Global Health Learning Community Steering Committee developed the interview guide using an iterative process. Interviewers conducted interviews in English; these interviews were audio-recorded and transcribed for analysis. Transcripts were de-identified, and audio and transcription files were stored in a secure, encrypted Cloud storage file.

### Data analysis.

Descriptive statistics were used to describe respondents’ basic demographic characteristics. The existence of a bilateral partnership was recorded as “yes,” “limited,” or “no” responses. A “yes” answer indicated that within the study participant’s GHP, trainees and faculty from both the international and United States-based institutions traveled to the other site for GH experiences. A “limited” partnership had one or two faculty from the international site who would occasionally visit the United States-based institution for training, whereas the international partner regularly hosted trainees and faculty from the United States-based institution. A “no” answer indicated that the international partner hosted United States trainees and faculty, but none of their own faculty, staff, or trainees traveled to the United States site.

We analyzed interview transcripts for themes regarding participants’ perspectives and insights regarding how the COVID-19 pandemic impacted their GHPs with United States-based institutions. The *a priori* codes were created from the interview guide as a starting point for analysis. Two authors (A. N., M. A. E.) independently performed line-by-line coding using the qualitative analysis software Dedoose.^9^ Three authors trained to perform qualitative analysis (A. N., M. A. E., M. S. M.) independently coded open-ended responses and, using constant comparison, identified emerging themes^9^ that were triangulated to identify central concepts.^10,11^

The Indiana University Institutional Review Board approved this project as an exempt study.

## RESULTS

Eighty-four United States partners completed the initial query collecting e-mail addresses for their corresponding international partners. Forty-five international partners fulfilled the inclusion criteria and were contacted to enroll in the study. Of these, 14 potential participants expressed willingness to perform an interview and 10 participants completed an interview (response rate, 10/14). Of the 31 who did not respond to the study team, one was affiliated with a ministry of health and the remaining 30 were affiliated with academic health centers or universities. Interviews lasted an average of 30 to 45 minutes. Half of the partnerships were less than 5 years in duration and most (7/10) had some degree of bilateral exchange (Table 1). All participants expressed COVID-19-related stress; none of the hospital systems was overwhelmed at the time of the interview, but all encountered challenges in terms of COVID-19 mitigation and expressed worry for impending high case burdens.

**Table 1:** Characteristics of study participants

Variable	n (%)
Sex	
Female	4 (40)
Male	6 (60)
Region of institutions	
Central and South America	5 (50)
Sub-Saharan Africa	4 (40)
Southeast Asia	1 (10)
Number of partnerships*	
One	4 (40)
Multiple	6 (60)
Duration of primary partnership	
< 5 years	5 (50)
5–10 years	3 (30)
> 10 years	2 (20)
Presence of bilateral exchange	
Yes	3 (30)
Limited	4 (40)
No	3 (30)

* This number indicates the number of partnerships in which the international institution, represented by the study participant, engages.

We identified four emerging themes from the data: imbalanced decision-making regarding activities within partnerships during COVID-19; limited communication during COVID-19; use of technology within partnerships during COVID-19; and future outlooks regarding partnerships after COVID-19.

### Imbalanced decision-making regarding activities within partnership during COVID-19.

When the COVID-19 pandemic began, most international partners in our study had United States trainees from their partnership onsite or their own trainees were abroad at United States partner sites. Regarding decision-making, United States and international partners had differing approaches to their off-site trainees as the pandemic progressed. For United States trainees onsite at international partner sites, the United States partner decided when they should to return to the United States and relayed their decision to their international partners. For international trainees onsite at United States partner sites, the international partner asked their trainees to contact their country embassy, not the United States institution, for decisions related to evacuation. For the majority (4/6) of the participants interviewed with trainees at their sites at the start of the pandemic, the United States-based institution made decisions regarding the level of ongoing engagement. For example, one participant stated:

“When the pandemic began, the U.S. closed [their] airports and said students had to be back [in the U.S.], and we [arranged for them to return.]. That is the way the U.S. works in the world.”- Participant, bilateral partnership of more than 10 years

Although this decision-making was unbalanced, it was understood and well-accepted by participants, especially because their partners based in the United States were liable and responsible for the safety of their own trainees.

Locally, international partner sites also had to make decisions regarding United States trainees and faculty traveling within their country during their rotation. A few sites allowed United States trainees and faculty to travel to rural sites. However, representatives from these rural sites asked the United States visitors to not travel to their sites because they feared their presence may lead to increased COVID-19 spread and limited the capacity to contain an outbreak. The international sites reported that they respected the rural sites’ requests, and that this did not incite conflicts with the United States visitors or partners.

Minimal conflicts between United States-based institutions and international partners were reported by interviewees regarding decision-making during the COVID-19 pandemic, and those that were identified occurred when international sites were still hosting United States trainees at the start of the pandemic. One example of such a conflict involved the necessity to quarantine. When this incident occurred, United States trainees refused an international site’s request to quarantine for 2 weeks upon arrival, thus prompting the international partner to request the United States-based partner to intervene. Ultimately, the trainees were asked by their home institution to return to the United States soon after the incident.

### Limited communication during COVID-19.

Some (5/10) participants noted that communication between partners decreased during the pandemic, and all expressed understanding regarding why this occurred. Some of participants from smaller partnerships with fewer staff reported feeling grateful for this temporarily decreased engagement because they were struggling to manage local COVID-19 cases and did not have time to engage with partners.

Of those who noted decreased communication, a few also noted that communication had shifted to address pandemic-related priorities, such as receiving COVID-19 treatment guidelines from their United States-based partner institution. None of the international institutions reported sharing resources with their partner based in the United States. Several participants noted that although formal partnership communication frequency had decreased, personal communications had increased, with partners checking on each other’s wellbeing. Some described that they wanted more formal communication, but respondents clarified that this desire had preceded the pandemic.

### Use of technology within partnerships during COVID-19.

Few participants reported transitioning to virtual engagement with their United States-based partner institution after travel restrictions were placed; however, most were interested in this possibility. A few participants representing unidirectional partnerships that primarily host United States trainees noted that their institutions were developing content for United States trainees to benefit from local expertise using a virtual platform, and that they hoped to be able to generate revenue by doing this to help support their organization during the pandemic. One participant noted that technology could facilitate more virtual engagement and decrease the need for in-person travel.

“[After the pandemic] most of the conversations might be continued online and then, once in a while, they may visit… because it is more convenient and then it is less expensive.” – Participant, unilateral partnership of less than 5 years

Some participants expressed hesitation toward fully converting to virtual collaborations because they would not adequately replace the immersive in-person experience.

“I can listen to a lecture from a U.S. professor through the phone and okay that’s great. He’s the great neurologist, and that’s good. [However] I think the most valuable experience from international rotations is actually going abroad, living in a different area, getting away from home.” Participant, bilateral partnership of less than 5 years

Only one participant noted that unreliable Internet would prohibit meaningful virtual collaborations; however, another participant had connectivity issues during the interview that required the responses to be sent via e-mail to the interviewer.

### Future outlooks regarding partnerships after COVID-19.

Although many participants were hopeful that their relationships with their United States partners would be restored after the COVID-19 pandemic, this hope was often mixed with worry about the relationship continuing at the same level of engagement. Participants from institutions with multiple international collaborations differentiated in that longer-standing partnerships were more likely to continue after COVID-19 than more recently formed partnerships. Fewer visits from United States trainees and faculty were anticipated, and this was a prominent worry for organizations with unilateral partnerships that depend on program fees from visiting United States trainees.

“We are a small organization. We got to even smaller [during the pandemic]. We are taking very basic roles and trying to just keep the channels of communication open. But we are not actually doing any teaching, research, or community engagement, which were our 3 pillars. So that is how it has affected us. Obviously, that has huge budget, need, and goal implications for us.” – Participant, unilateral partnership of less than 5 years

“We know [partner] is a very wealthy organization. They are very generous and helping us in every way they can, but we know the emergency changed the world and we have an economic crisis worldwide, so we know we are not the priority, the priority is to help locally first. So, my concern maybe is to stop receiving some help from them.” Participant, bilateral partnership of 5–10 years

“But for example, we have a relationship with [partner] for about 25 years but [partner] use to sign an agreement with us. The agreement [at present] is only verbal agreement. I don’t know what is the next step with the end of this pandemic.” Participant, bilateral partnership of more than 10 years

A few participants recognized the benefits that their institutions provide to their United States-based partner institution and believed these benefits would endure and result in continued engagement after the pandemic. One participant understood how the partnership benefited the United States-based partner institution; therefore, that participant was confident in the future partnership:

“There is no way they can be called an international organization if they don’t relate with other international countries”- Participant, unilateral partnership of 5–10 years

Some participants expressed future desires for their partnerships that stemmed primarily from pre-existing interests and not from the pandemic. The most prominent of these interests was to have their United States-based partner institution host more of their trainees and faculty. One participant described the desire for more meaningful and structured relationships with the partner:

“I’d love to do something more meaningful for partnerships. It’s good to go for clinical rotations for 1–2 months … but I don’t like the idea of just signing a [Memorandum of Understanding], sending 2 students that way and sending 2 students this way. Yeah, we can do that, but it would be interesting to have professors, faculty members engaging with each other … it can be research or education … we need to deepen and strengthen our relationships”- Participant, bilateral partnership of less than 5 years

Although these common core themes were identified within the dataset, we did not achieve thematic saturation because of the limited sample size.

## DISCUSSION

Within this qualitative study, we drew from perspectives and insights regarding international partners to understand how the COVID-19 pandemic impacted and continues to impact GHPs. Despite the limited number of participants, the interviews provided critical considerations for United States-based partner institutions. Overall, international partners were positive about their experiences since the pandemic began. However, it appears that much of the decision-making during the pandemic was unilaterally led by the United States partner, and several participants expressed worry about the state of these partnerships after COVID-19. Many were open to using technology to help continue education and training, although some limitations may arise, such as unstable Internet and the lack of immersive interactions. A few participants expressed the desire for stronger communication and robust bilateral exchanges that would grow more meaningful relationships, but these challenges existed before COVID-19 and were highlighted because of the pandemic.

Undoubtedly, GHPs will look different during the era after the COVID-19 pandemic. One promising avenue for this change would be increased virtual GH engagements. Most participants reported adequate access to Internet, and many expressed an interest in pursuing virtual options for GH in the future. This does suggest possibilities for the future bilateral exchange of information between institutions. During the COVID-19 pandemic, many organizations launched virtual webinar series to continue dialogue about important GH topics during the pandemic, such as John Hopkins Global COVID Webinars and the American Academy of Pediatrics Global Webinar series.^12,13^ Organizations such as Child Family Health International have created repositories of virtual opportunities for GH learning,^14^ with the primary target of these resources being United States-based individuals interested in GH and unable to engage internationally. Furthermore, some partnerships have embraced bilateral virtual engagements, such as telemedicine consults, practicums, or virtual rounds.^15^ Future studies are needed to understand the current status, preference, and capacity of virtual learning for both the United States and international partners, and one such study is currently being conducted by Umphrey et al.^16^

Most communication between partnering institutions appeared to be positive, with some participants describing that communication during the height of the pandemic focused primarily on the health and welfare of their partners. However, a few experiences may warrant further consideration. One such experience was the refusal by United States trainees to self-quarantine after arriving to the international host country. During the early days of the pandemic, limited understanding about and formal guidance regarding COVID-19 brought great uncertainty and contributed to challenges in communication and decision-making. A guiding principle for ethical and sustainable GHP^2^ is to avoid adverse effects of trainee involvement within the partnership. This principle should have been particularly emphasized at the start of this wide disruption. Similarly, early during the pandemic, participants described instances when their organization stopped sending United States trainees and faculty to rural sites within their country because of concerns that COVID-19 might be transmitted within settings without appropriate healthcare capacity. Although most international travel halted during the peak of the pandemic, United States-based institutions now have an opportunity to learn from and work alongside their international partners to modify predeparture curricula to include appropriate health precautions, define mutual institutional risk management processes, and develop quality cultural humility resources. Furthermore, GH education now has the opportunity to restructure its relationship and address supremacy at a systemic level.^17^ A foundation of mutual respect and open communication must be further cultivated during the postpandemic era, and the tendency for unilateral decision-making that was experienced within GHPs during COVID-19 must be avoided. Future research and discussions should focus on how to optimize communication and establish mutual, equitable exchanges within GHPs.

The results of this study are limited by the small sample size, resulting in a lack of saturation of themes and a lack of comprehensive representation from international partner institutions. We recognize that we could not capture broad perspectives and insights during this global pandemic and that several limitations resulted in a small sample size. For example, the recruitment for this study occurred during the COVID-19 pandemic; to reach international partners, we relied on United States-based partners to share corresponding e-mail addresses. Prospective participants who were healthcare providers could have been overwhelmed with clinical or administrative responsibilities and, understandably, unable to participate. This is supported by the fact that the majority of eligible participants who were nonrespondents were affiliated with academic health centers or universities. Furthermore, it is possible that only international partners with strong ongoing relationships with their United States-based partner completed the demographic survey and felt encouraged to participate. We also speculated that prospective participants available for an interview represent communities or institutions less impacted by the pandemic at that moment or had adequate resources to engage (i.e., Internet connectivity). It is also possible that social desirability bias may have been inadvertently introduced within this study because the preservation of supportive relationships is a likely priority for many institutions at this time. We attempted to minimize this bias by utilizing interviewers unrelated to the study participant’s United States-based institution and by using neutral, open-ended questioning. We also allowed participants to choose not to name their United States-based partner institution to further preserve anonymity. Although all participants felt comfortable with English, it may have not been the primary language for many. The results of this study were also influenced by the specific time period of data collection during the pandemic. If data collection had occurred later during the pandemic, when more was known about the virus and immunizations were available, then it is possible that experiences of the GHP may have differed. Despite these limitations, our intention was to acknowledge the experiences and not diminish those of others. We feel that participants of this study contributed meaningful perspectives and insights regarding how GHPs can strengthen collaborations in the future.

From the GHPs represented in this qualitative study, we found that most international partners are hopeful but also worried for the future of their international collaborations because of the COVID-19 pandemic. Although not representative of all GHPs, the participants’ valuable information provided the United States and international partners with key considerations for discussion and strategic development. As we begin to transition past the acute phase of the COVID-19 pandemic, United States-based institutions have an opportunity to engage in more equitable decision-making, strengthen communication, and collaborate innovatively with their international partners. We will continue to encounter global uncertainties and challenges in the future. Both the United States and international partner institutions should be pushed now more than ever to recognize their dependency on each other, create clear policies for trainee and faculty exchange and safety, and create meaningful deeper relationships while working toward shared health-related goals. This is our way forward, together.
